# Changes in the epidemiological profile of Bordetella pertussis infection

**DOI:** 10.36416/1806-3756/e20260401

**Published:** 2026-09-14

**Authors:** Francine Bester Damian, Maria Fernanda Goncalves Meirelles Fernandes, Ana Barbosa Zocolotti, Marcos Otávio Brum Antunes, Leonardo Araújo Pinto

**Affiliations:** 1. Grupo de Pesquisa em Epidemiologia e Genética das Doenças Respiratórias da Infância, Pontifícia Universidade Católica do Rio Grande do Sul - PUCRS - Porto Alegre (RS) Brasil.; 2. Programa de Pós-Graduação em Medicina - Pediatria, Escola de Medicina, Pontifícia Universidade Católica do Rio Grande do Sul - PUCRS - Porto Alegre (RS) Brasil.

## INTRODUCTION

Pertussis is a vaccine-preventable respiratory infection caused by *Bordetella pertussis* that has re-emerged despite decades of immunization programs.^1^ ^,^ ^2^ Transmission occurs mainly through respiratory droplets, and adolescents and adults with mild, atypical, or prolonged cough may infect infants who have not completed the primary vaccination schedule.^2^ Although infants remain at greatest risk of hospitalization, complications, and death, pertussis is increasingly recognized across a broad range of age groups.^2^ ^,^ ^4^

Its contemporary epidemiology reflects the interaction of multiple factors, including waning immunity, differences between vaccine formulations and schedules, pathogen adaptation, incomplete vaccination, and vaccine hesitancy.^1^ ^,^ ^2^ The relative contribution of these factors varies according to population immunity, diagnostic capacity, surveillance practices, and vaccination coverage.

## EPIDEMIOLOGICAL TRENDS OF PERTUSSIS

After the marked decline following vaccine introduction, pertussis became endemic with recurrent epidemic peaks. These cycles often occur every three to five years and reflect the accumulation of susceptible individuals, changes in population immunity, and continued transmission.^2^ ^,^ ^5^ Surveillance data are influenced by healthcare-seeking behavior, clinical suspicion, laboratory access, case definitions, and completeness of notification.

Figure 1 presents the annual number and population-based rate of reported pertussis cases in Brazil from 2001 to 2025. Between 2001 and 2010, annual counts remained comparatively low, ranging from 592 cases in 2010 to 1,403 in 2008. A marked increase began in 2011, followed by 5,402 cases in 2012, 6,381 in 2013, and a peak of 8,498 in 2014. The corresponding rate reached approximately 4.2 cases per 100,000 population, the highest value in the series.

**Figure 1:**
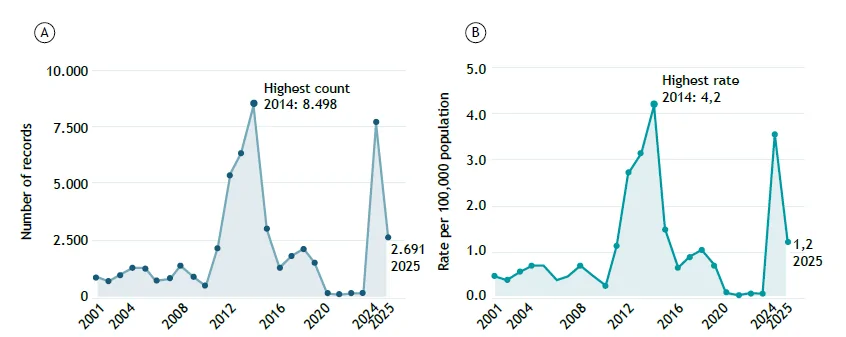
Temporal trend in reported pertussis cases in Brazil, 2001-2025. Panel A shows the annual number of reported cases, and Panel B shows the annual rate per 100,000 population. Population denominators were obtained from IBGE/SIDRA.

After 2014, reports decreased to 3,051 in 2015 and 1,309 in 2016. A smaller rise reached 2,171 cases in 2018, followed by another decline in 2019. The lowest levels occurred during and immediately after the main COVID-19 pandemic period: 232 cases were reported in 2020, 159 in 2021, 238 in 2022, and 222 in 2023. Rates remained below 0.2 per 100,000 population. This decline probably reflected reduced respiratory transmission, changes in healthcare use, underdiagnosis, and disruptions to surveillance and routine healthcare services.^2^ ^,^ ^4^

In 2024, Brazil recorded 7,748 reported cases, approximately 3.6/100,000 population, representing a 34.9-fold increase from 2023. A separate national analysis also documented a marked resurgence and a greater proportion of cases among people aged 15 years or older.^4^ In 2025, reports decreased to 2,691 cases and 1.2/100,000 population. These values remained substantially above those observed from 2020 to 2023. The national series therefore shows two major peaks, in 2014 and 2024, separated by exceptionally low levels of notification during the pandemic years.

## CHANGES IN THE DEMOGRAPHIC PROFILE OF THE DISEASE

The resurgence has been accompanied by increased recognition of pertussis among older children, adolescents, and adults.^2^ This does not mean that the burden has shifted away from infancy. Infants, particularly those who are unvaccinated or incompletely vaccinated, remain at greatest risk of severe disease, hospitalization, and death. Older age groups remain epidemiologically important because mild or atypical infection may delay diagnosis and facilitate transmission.^2^

In Brazil, the 2024 resurgence included an increase in cases among individuals older than 15 years of age, although children continued to account for an important proportion.^4^ Clinical suspicion should extend to adolescents and adults with prolonged or paroxysmal cough, especially those in contact with pregnant women or infants.

Countries with high national vaccination coverage have not been exempt from outbreaks.^2^ Average coverage may conceal geographic or social clusters of delayed vaccination or incomplete primary vaccination schedules.^3^ The Brazilian program also differs from those using exclusively acellular childhood vaccines: the routine schedule includes three doses of a whole-cell pertussis-containing pentavalent vaccine, followed by two diphtheria and tetanus toxoids and pertussis (DTP) boosters.^5^ Explanations centered on the replacement of whole-cell vaccines by acellular vaccines should therefore not be transferred directly to Brazil.

## FACTORS ASSOCIATED WITH PERTUSSIS RESURGENCE

Waning immunity is a major contributor to recurrent transmission. Neither natural infection nor vaccination provides lifelong protection.^2^ Immunity after acellular vaccination may decline more rapidly than after schedules including at least one whole-cell dose.^2^ This evidence is especially relevant to countries using exclusively acellular schedules, but it also shows that previous vaccination or infection does not completely exclude the possibility pertussis in older age groups.

Pathogen adaptation may also contribute to transmission. Genetic changes in *B. pertussis*, including the *ptxP3* promoter genotype and pertactin-deficient strains, have been associated with increased toxin production or selective advantages in populations vaccinated with acellular vaccines.^2^ These findings support a multifactorial explanation for resurgence but do not indicate that vaccination has become ineffective.

Incomplete vaccination and vaccine hesitancy may increase susceptibility. A systematic review found a higher risk of pertussis among unvaccinated or undervaccinated infants and children and supported the protective role of maternal vaccination.^1^ Local pockets of underimmunization may sustain transmission even when national coverage appears high.^3^ Pandemic-related disruptions to routine immunization may also have widened immunity gaps.^2^

## IMPLICATIONS FOR SURVEILLANCE AND CONTROL STRATEGIES

Vaccination remains the central prevention strategy. In Brazil, priorities include timely completion of the childhood series and boosters, dTpa vaccination during every pregnancy, and vaccination of other eligible groups according to national recommendations.^5^ Maternal vaccination is particularly important because it provides passive protection to infants during the first months of life.

Laboratory confirmation can be performed by culture of a nasopharyngeal specimen or by real-time polymerase chain reaction; culture remains the reference standard in national guidelines.^5^ Diagnostic capacity is insufficient if pertussis is not considered clinically, particularly in adolescents and adults with persistent cough or in household contacts of infants.

The true burden is probably underestimated because mild and atypical cases may not seek care, may receive alternative diagnoses, or may not undergo testing.^2^ Surveillance should integrate notifications, laboratory results, vaccination history, age distribution, and geographic information. Ecological evidence from King County suggests that local coverage, household size, and median income may be associated with patterns of pertussis occurrence.^3^ These findings cannot be directly generalized to Brazil, but they support analyses at state, municipal, and submunicipal levels.

The Brazilian resurgence demonstrates that sustained control requires timely vaccination, equitable access, sensitive surveillance, prompt diagnosis, and protection of infants through maternal and childhood immunization. The increase in 2024, followed by a lower but substantial number of reported cases in 2025, supports continued monitoring rather than assuming that the resurgence was an isolated event.
